# Rainfall and Temperature Explain Colony Variation in Echolocation Calls of the Intermediate Horseshoe Bats (*Rhinolophus affinis*)

**DOI:** 10.1002/ece3.73485

**Published:** 2026-04-08

**Authors:** Dongge Guo, Lixian Wang, Chong Zhang, Siyuan Dong, Fang Wang, Bo Luo, Sen Liu

**Affiliations:** ^1^ College of Life Science Henan Normal University Xinxiang China; ^2^ Observation and Research Field Station of Taihang Mountain Forest Ecosystems of Henan Province Xinxiang China; ^3^ College of Physical Education Henan Normal University Xinxiang China; ^4^ Key Laboratory of Southwest China Wildlife Resources Conservation of Ministry of Education China West Normal University Nanchong China

**Keywords:** Chiroptera, echolocation call, ecological selection, geographic variation, *Rhinolophus affinis*

## Abstract

Geographic variation in acoustic signals of animals is shaped by both environmental conditions and morphology related to vocal production. However, the drivers of call variation among colonies at a landscape scale remain largely uncertain. This study aimed to assess the determinants of colony‐level variation in echolocation calls of intermediate horseshoe bats ( *Rhinolophus affinis* ) from the southern and northern regions of the Funiu Mountains in central China. We recorded echolocation calls emitted by 129 resting adult bats from eight cave‐dwelling colonies and measured the spectro‐temporal parameters of their calls. We obtained forearm lengths of bats, climate variables, and the normalized difference vegetation index to evaluate their effects on echolocation call variation among colonies. Our analyses showed that the duration and peak frequency of echolocation calls varied among colonies and between sexes. Males produced echolocation calls of longer durations and lower peak frequencies relative to females. Monthly mean precipitation was positively associated with the peak frequency of echolocation calls in males. The monthly mean temperature was negatively associated with the peak frequency of echolocation calls in females. In both males and females, call duration and peak frequency were not significantly affected by forearm length, altitude, or normalized difference vegetation index. The structural equation model further indicated that monthly mean precipitation and temperature had direct effects on call frequency of males and females, respectively. These results provide correlative evidence in favor of the sensory drive hypothesis, indicating that rainfall and ambient temperature shape echolocation call divergence in horseshoe bats at the landscape scale.

## Graphical abstract

We investigated how environmental factors and body size drive colony‐level variation in echolocation calls of *Rhinolophus affinis* at a landscape level. Our analyses revealed a positive correlation between monthly mean precipitation and peak frequency of echolocation calls in males. The peak frequency of echolocation calls in females was negatively associated with monthly mean temperature. These results indicate that rainfall and temperature shape echolocation call divergence in horseshoe bats at the landscape scale.

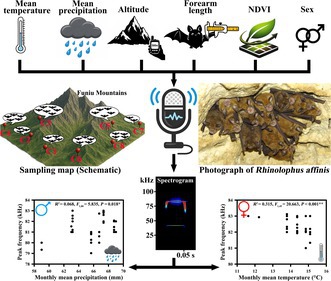

## 1 Introduction

Acoustic signals mediate multiple life history events essential for animals' survival and reproductive success, including foraging, predator avoidance, mate attraction, and territory defense (Wilczynski and Ryan 1999; Bradbury and Vehrencamp 2011). A growing body of literature indicates that acoustic signals exhibit geographic variation across invertebrate and vertebrate animals, encompassing insects (Lampe et al. 2014), anurans (Ryan et al. 1990), birds (Nemeth et al. 2013), and mammals (Mitani and Stuht 1998; Jiang et al. 2015). Such variation in acoustic signals may arise due to selective pressures imposed by the local environment, wherein environmental factors such as temperature and humidity directly influence sound transmission (Morton 1975; Podos and Warren 2007; Wilkins et al. 2013). Meanwhile, the sound‐production apparatus and associated morphological traits may also contribute to geographic divergence in acoustic signals among some animals (Ryan and Kime 2003). So far, the relative importance of environmental factors and morphology underlying geographic variation in acoustic signals remains largely unknown in most animal taxa.

Constant‐frequency (CF) bats primarily use echolocation calls for spatial orientation and foraging, including species in the families Rhinolophidae, Hipposideridae, Rhinonycteridae and Parnell's mustached bat ( *Pteronotus parnellii* ) (Fenton et al. 2012; Jones and Teeling 2006; Jones and Holderied 2007; Luo et al. 2019). CF bats are high duty cycle echolocators that emit long constant‐frequency echolocation calls, typically lasting from 20 to 100 ms. These calls are accompanied by a downward broadband sweep, an upward broadband sweep, or both (Fenton et al. 2012; Luo et al. 2019). A long call at constant frequency can increase signal energy, and their echoes rapidly trigger neuronal filters that are tuned to narrowband signals, thus facilitating the detection of fluttering insects (Kalko and Schnitzler 1998; Schnitzler and Kalko 2001). A broadband sweep can activate more neuronal filters, improving the accuracy of range and angle determination (Kalko and Schnitzler 1998). Therefore, a combination of long CF component and broadband sweep maximize the performance of prey detection and localization in highly cluttered habitats (Schnitzler and Kalko 2001). CF bats face auditory masking effect when their own echolocation calls and clutter echoes overlap with the echoes from prey, which hinders their echolocation ability (Kalko and Schnitzler 1998). To prevent auditory masking, CF bats employ Doppler shift compensation to adjust the frequency of prey echoes within the sensitive frequency range of the auditory fovea in the cochlea and the sharply tuned neurons in the central auditory system (Schnitzler 1973; Schuller and Pollak 1979; Schnitzler and Denzinger 2011).

Previous studies have shown that morphological traits associated with sound production, in conjunction with environmental factors, contribute to geographic variation in echolocation calls among some CF bats (Jiang et al. 2015; Jacobs et al. 2017; Russo et al. 2018). The sensory drive hypothesis proposes that geographic variation in echolocation calls of CF bats results from adaptations to local ecological conditions, since climate and habitat structure influence sound transmission and thereby shape the acoustic environment in which bats operate (Jiang et al. 2015; Mutumi et al. 2016; Maluleke et al. 2017). In particular, echolocation calls of bats undergo heightened attenuation in warm and moist environments, since atmospheric absorption intensifies with rising ambient temperature and humidity (Goerlitz 2018; Lawrence and Simmons 1982). Moreover, rainfall can produce broadband background noise that may overlap spectrally with echolocation calls of bats (Ma et al. 2022), and vegetation structures cause clutter echoes through sound reflection and scattering (Schnitzler et al. 2003). Therefore, rainfall and vegetation structures may impair the performance of bat echolocation through auditory masking and crowding effects. Indeed, Jacobs et al. (2017) found that higher ambient temperature caused a decline in resting frequency of echolocation calls in Geoffroy's horseshoe bat ( *Rhinolophus clivosus* ) under conditions of moderate to high humidity. In Noack's roundleaf bat ( *Hipposideros ruber* ) and Least horseshoe bat ( *R. pusillus* ), resting frequency of echolocation calls is influenced by annual mean rainfall (Guillén et al. 2000; Jiang, Metzner, et al. 2010). In Cape horseshoe bats ( *R. capensis* ), resting frequency of echolocation calls was positively related to vegetation index around colony roosts (Odendaal et al. 2014). In addition, the acoustic space available to some echolocation callers is constrained by their sound‐production apparatus (Jones 1999; Yoshino et al. 2006). The morphological constraint hypothesis posits that the size and structure of sound‐producing apparatus influence spectro‐temporal parameters of echolocation calls in bats, leading to inter‐colony call variations that correspond to morphological differences (Jiang, Liu, et al. 2010; Russo et al. 2018). In Okinawan least horseshoe bats ( *R. pumilus* ), colonies with smaller body sizes produced echolocation calls of higher peak frequency compared to colonies exhibiting larger body sizes (Yoshino et al. 2006). Similarly, larger Great leaf‐nosed bats ( *H. armiger* ) uttered echolocation calls at lower frequencies than smaller individuals from allopatric colonies (Lin et al. 2015). Collectively, these previous studies uncovered the patterns and causes of geographic variation in bat echolocation calls at a broad geographical scale of more than 100 km. For clarity, we distinguish three spatial scales in this study. Broad geographical scale refers to distances greater than 300 km and encompasses variation among widely separated regions. Landscape scale denotes spatial extents of approximately 20–100 km and captures variation among nearby colonies within a region. Local intra‐colony scale refers to distances less than 10 km and encompasses fine‐scale variation within a single colony. These scales are expected to differ in their dominant selective pressures (Russo et al. 2018). Although call variation has been relatively well characterized at broad and local scales, the determinants of variation in echolocation calls among colonies at the landscape‐level scale (i.e., within 100 km) remain poorly understood.

The Intermediate horseshoe bats ( *Rhinolophus affinis* ), a CF member of the family Rhinolophidae, typically roosts in caves and other subterranean habitats. This broadly distributed taxon occurs across South Asia and mainland Southeast Asia, including the southern Himalayas and parts of China. In China, *R. affinis* is predominantly distributed south of the Qinling‐Huaihe Line, including Henan, Hunan, Hubei, Sichuan, Fujian, Jiangxi, Yunnan, Guangdong, Guangxi, and Hainan provinces (Furey et al. 2020). The species is currently divided into three subspecies, namely *R. affinis himalayanus* , *R. affinis hainanus* , and *R. affinis macrurus* . Previous genetic work suggests that the mainland subspecies *R. affinis himalayanus* migrated to Hainan Island, where it formed the subspecies *R. affinis hainanus* . Subsequently, *R. affinis hainanus* recolonized the mainland, leading to the formation of *R. affinis macrurus* (Mao et al. 2010, 2014). The peak frequency of *R. affinis* echolocation calls in China ranges from 75 to 86 kHz (Jiang et al. 2008; Luo et al. 2019). In this study, we recorded echolocation calls from resting male and female *R. affinis* across eight colonies located in the southern and northern regions of Funiu Mountains, a transitional zone between the Palearctic and Oriental biogeographic realms in China. These colonies belong to the mainland subspecies, *R. affinis himalayanus* .

The goal of this study was to assess the determinants of colony‐level variation in echolocation calls of resting intermediate horseshoe bats on the landscape scale. First, if climate‐mediated ecological selection drives colony variation in echolocation calls, we predict that call duration and peak frequency would be negatively associated with ambient temperature, humidity, and precipitation. Second, if habitat structure drives colony variation in echolocation calls, colonies in more structurally complex habitats would exhibit call modifications that mitigate clutter and scattering. Third, if morphological constraint is responsible for colony variation in echolocation calls, we predict that call duration and peak frequency would be negatively associated with body size. Fourth, if elevation influences colony variation, colonies at higher elevations would emit longer calls with lower peak frequencies than colonies at lower elevations.

## 2 Materials and Methods

### 2.1 Study Area and Sample Collection

**FIGURE 1:**
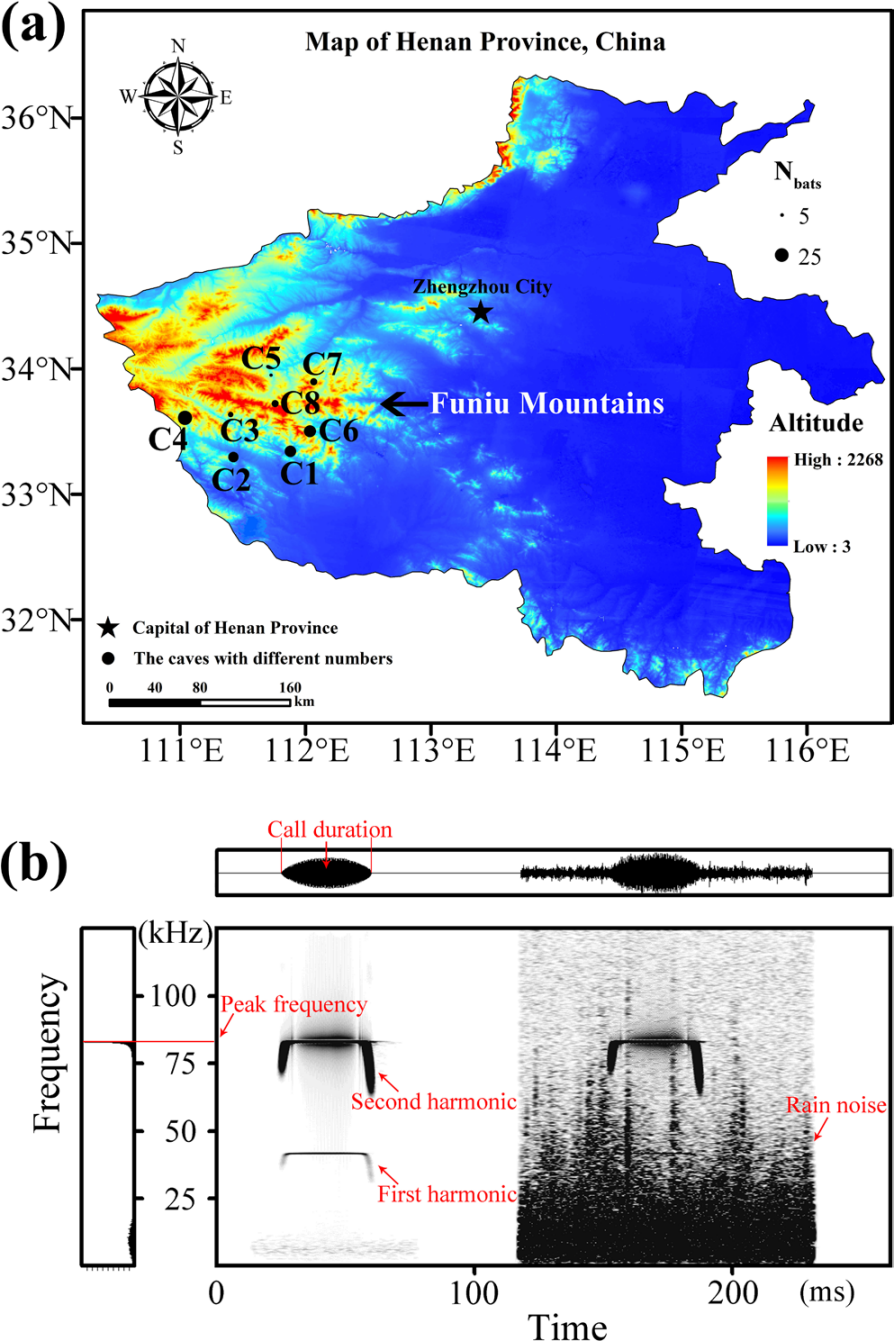
Roost locations and echolocation call characteristics of *Rhinolophus affinis* . (a) Roost locations (C1–C8) of eight colonies in the Funiu Mountains. Solid black circle size indicates the sample size of each colony. C1: Tianxin Cave; C2: Yunhua Cave; C3: Jiulong Cave; C4: Longsangong Cave; C5: Shuilian Cave; C6: Xianren Cave; C7: Xiapu Mine; C8: Kafang Mine. (b) Spectrogram of an echolocation call with overlapped local rain noise. The central frame displays the sonogram, the upper frame shows the oscillogram, and the left frame displays the power spectra. Red labels and lines indicate measured acoustic parameters and reference lines.

The field study was conducted in the Funiu Mountains of central China. This mountain range extends approximately 400 km and lies near the Qinling‐Huaihe Line, a major biogeographic boundary. Noticeable microclimatic differences were observed between the northern and southern sections of the Funiu Mountains: the northern region falls within the warm temperate zone, whereas the southern region belongs to the subtropical zone. In this region, we surveyed eight colonies of *R. affinis* roosting in natural and man‐made caves. Four colonies (C1–C4) were located in the southern region of the Funiu Mountains, while the remaining four colonies were situated in the northern region (C5–C8; Figure 1a). Two colonies (C3 and C5) were composed exclusively of male *R. affinis* , while the remaining six colonies comprised individuals of both sexes. Each colony's roost was surrounded by some trees and shrubs. Inter‐colony distances ranged from 23 to 101 km (Figure 1a).

We captured *R. affinis* from eight colonies at their respective roosts from June to July 2024, corresponding temporally with the pregnancy period in female bats. A mist net was deployed at the entrance of each cave occupied by *R. affinis* 30 min before local sunset. In total, 129 individuals (82 ♂ and 47 ♀) were captured from eight colonies (Table S1). Although some movement among nearby roosts cannot be excluded, horseshoe bats generally show strong roost fidelity and limited dispersal during the breeding season, so the eight colonies in our study can be treated as approximate independent sampling units for landscape‐scale comparison (Lewis 1995; Budinski et al. 2019). Each captured bat was placed individually in a soft, breathable cloth bag. The age of each bat was determined based on the degree of epiphyseal fusion and fur coloration (Kunz and Fenton 2003). Only adults were retained for echolocation call recording and morphological measurements, whereas juveniles were immediately released at their roosts.

### 2.2 Acoustic Recording and Analysis

All captured bats were transferred into temporary field stations near the bat roost within 30 to 60 min after capture. Each experimental bat was then placed in a metal cage (70 cm × 40 cm × 40 cm) for a brief acclimation. To avoid Doppler shift effects, echolocation calls were recorded for each bat until it remained motionless inside the cage, using an ultrasonic recorder (UltraSoundGate 116; Avisoft Bioacoustics, Berlin, Germany) connected to a laptop. We focused on resting echolocation calls because, in horseshoe bats, the resting frequency is closely linked to the component used for Doppler shift compensation and prey detection, making it an ecologically meaningful and standardized measure for comparing colonies and sexes. The ultrasonic recorder was operated at a sampling frequency of 250 kHz with 16‐bit resolution. A condenser microphone (UltraSoundGate CM16, Avisoft Bioacoustics, Berlin, Germany) was positioned ~0.5 m from the bat's head to optimize the signal‐to‐noise ratio. Recordings lasted for a duration of 1–6 min per bat. Upon the completion of acoustic data collection, all bats were released at their capture sites. The entire procedure for handling bats was approved by the Academic Ethics and Ethics Committee of Henan Normal University (permit ID: HNSD‐2024BS‐1105 and HNSD‐2024BS‐1230).

Echolocation calls of *R. affinis* with a signal‐to‐noise ratio above 10% were analyzed using Avisoft‐SASLab Pro version 4.40 (Avisoft Bioacoustics, Berlin, Germany) (Figure 1b). Acoustic analyses were performed with a Hamming window, 512‐point fast Fourier transform (FFT), 100% frame size, and 75% temporal overlap. Because call duration depends on pulse composition, we selected 15 single‐pulse calls from each bat for analysis. The initial 10 echolocation calls emitted by *R. affinis* were excluded from the analysis because horseshoe bats adjust their call frequency to the resting frequency after a period of silence (Schuller and Suga 1976; Siemers et al. 2005). Call duration (i.e., the temporal interval between the onset and end of the call) was measured from the oscillogram, and peak frequency (i.e., the frequency of maximum energy) from the power spectrum. *R. affinis* emitted echolocation pulses consisting of at least two harmonics, with most of the energy concentrated in the second harmonic (Figure 1b). Therefore, only acoustic parameters of the second harmonic were analyzed. For each individual, mean values of call duration and peak frequency were calculated for subsequent analyses.

### 2.3 Quantification of Predictor Variables

Six predictor variables potentially related to echolocation call parameters in *R. affinis* were obtained: forearm length, body mass, monthly mean temperature, monthly mean precipitation, normalized difference vegetation index (NDVI), and altitude. For each bat, forearm length was measured using a digital caliper (Guanglu 111N‐102‐40, Guangxi, China), and its body mass was determined using an electronic balance (Melien MTB 500, Guangdong, China) (Guo et al. 2021). Geographical coordinates (i.e., latitude, longitude, and altitude) of each study site were recorded with a handheld GPS device (Garmin eTrex 221x, Jiangsu, China). Monthly mean temperature and precipitation for each colony roost, spanning the period from 1970 to 2000, were obtained from WorldClim v2.1 at a spatial resolution of 30 arc‐seconds using R package terra (Fick and Hijmans 2017). To characterize the habitat structure for each colony, we extracted the NDVI corresponding to their geographic coordinates from a daily gap‐free NDVI dataset (Li et al. 2024), using the package raster (Hijmans 2025). This dataset was derived from the National Oceanic and Atmospheric Administration daily NDVI data covering the years from 1981 to 2023, with a spatial resolution of 0.05° (~5.56 km). Although the relatively coarse resolution of NDVI may not capture fine‐scale habitat features that are relevant to bat echolocation, NDVI reflects the amount of chlorophyll in vegetation, and is widely used as an indicator of vegetation cover and above‐ground primary productivity (Carlson and Ripley 1997; Odendaal et al. 2014).

### 2.4 Statistical Analysis

Kolmogorov–Smirnov tests were used to assess whether echolocation call duration and peak frequency conformed to a normal distribution. The peak frequency was Box‐Cox transformed to satisfy the normality. All continuous variables were standardized using z‐scores to minimize the effects of scale differences. The two‐way analysis of variance (two‐way ANOVA) was applied to examine the effects of colony and sex on echolocation call parameters. Linear mixed models (LMMs) were used to evaluate the relationships among echolocation call parameters, forearm length, and environmental variables. Given significant sexual dimorphism in echolocation call parameters, separate LMMs were fitted for male and female bats using R package lme4 (Bates et al. 2015). Call duration and peak frequency were designated as the dependent variable, while forearm length, monthly mean temperature, monthly mean precipitation, NDVI, altitude, and their significant interaction based on likelihood ratio test were designated as predictor variables. Sampling date was regarded as a random effect. Multicollinearity among candidate predictors was assessed before model selection, and model residuals were examined to confirm that the assumptions of normality were met. Additionally, structural equation model (SEM, also referred to as path model) was conducted to assess the direct and indirect effects of significant microclimatic variables on echolocation call parameters using the package lavaan (Rosseel 2012). Monthly mean temperature and precipitation were regarded as exogenous variables, and peak frequency and forearm length were regarded as endogenous variables (Luo et al. 2019). The best‐fitting LMM and path models (Tables S3, S4) were selected based on Akaike's information criterion corrected for small sample sizes (AICc). LMMs for call duration and peak frequency in both male and female *R. affinis* were repeated using body mass as an alternative proxy for body size. The results based on forearm length as the proxy for body size are presented in the main text, whereas the corresponding results based on body mass are provided in the Supporting Information for reference (Table S3). All statistical tests were two‐tailed with a significance level set at 0.05, and means are given ± SE.

## 3 Results

### 3.1 Colony Variation in Echolocation Calls of Bats

**FIGURE 2:**
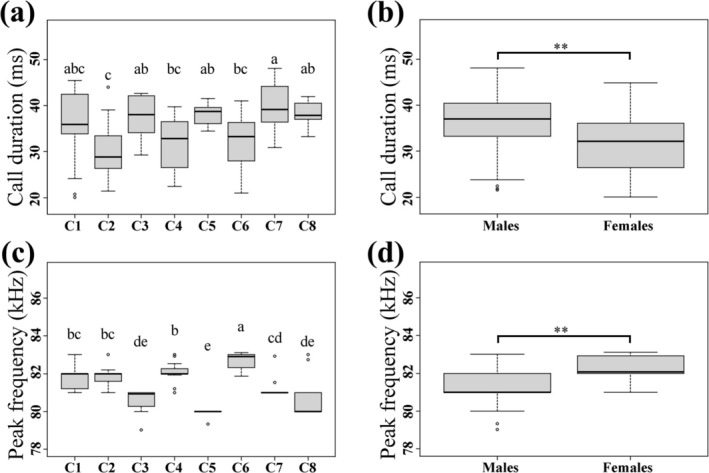
Variations in echolocation call parameters of *Rhinolophus affinis* . (a) Mean call duration among colonies. (b) Mean call durations between sexes. (c) Mean peak frequency among colonies. (d) Mean peak frequency between sexes.

The total recording duration for *R. affinis* amounted to 338 min, with an average of 2.62 min per bat. The duration and peak frequency of echolocation calls in *R. affinis* exhibited significant variations among colonies (two‐way ANOVA: call duration: *F*_7, 115_ = 4.29, *p* = 0.0003; peak frequency: *F*_7, 115_ = 26.90, *p* < 0.0001) and between sexes (two‐way ANOVA: call duration: *F*_1, 115_ = 18.42, *p* < 0.0001; peak frequency: *F*_1, 115_ = 127.09, *p* < 0.0001; Figure 2). There was a significant interaction between colony and sex for peak frequency (two‐way ANOVA: *F*_5, 115_ = 5.83, *p* < 0.0001), whereas no significant interaction was detected for call duration (two‐way ANOVA: *F*_5, 115_ = 1.72, *p* = 0.14). In general, males produced echolocation calls with longer duration (34.42 ± 6.79 ms) than females (30.10 ± 7.28 ms). The peak frequency of echolocations calls was lower in males (81.46 ± 1.06 kHz) relative to females (82.42 ± 0.43 kHz). At the colony level, the Xiapu Mine (C7) colony exhibited the longest call duration (40.15 ± 5.23 ms), followed by the colonies from Kafang Mine (C8), Shuilian Cave (C5), Jiulong Cave (C3), and Tianxin Cave (C1) (Figure 2a). The Yunhua Cave (C2) colony produced the shortest echolocation calls compared with other colonies (Figure 2a and Table S1). The Shuilian Cave (C5) colony had the lowest peak frequency (79.87 ± 0.30 kHz), followed by Jiulong Cave (C3) colony (80.60 ± 0.66 kHz), whereas the highest peak frequency occurred in the Xianren Cave (C6) colony (82.69 ± 0.42 kHz; Figure 2c and Table S2).

### 3.2 Determinants of Echolocation Call Divergence in Bats

**FIGURE 3:**
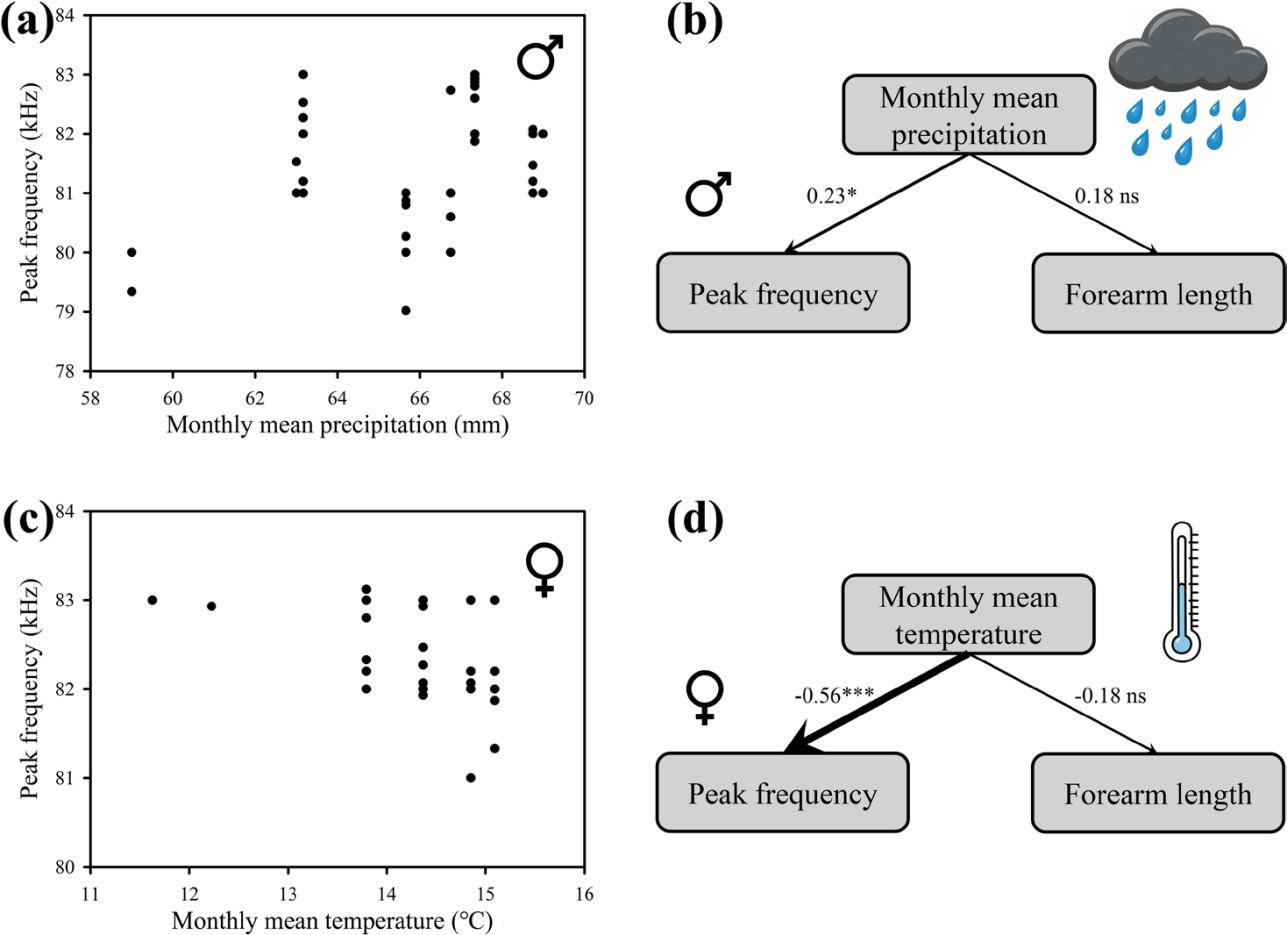
Relationships between predictor variables and peak frequency of echolocation calls in *Rhinolophus affinis* . (a) Relationship between monthly mean precipitation and peak frequency of echolocation calls in males. (b) Path diagram showing direct effects of the predictors on peak frequency of echolocation calls in males. (c) Relationship between monthly mean temperature and peak frequency of echolocation calls in females. (d) Path diagram showing direct effects of the predictors on peak frequency of echolocation calls in females. *p < 0.05, ***p < 0.001, ns: not significant.

In the best‐fitting linear mixed models (LMMs), monthly mean temperature (MMT) was the only predictor of call duration for both male and female bats (Table S3). Monthly mean precipitation (MMP) and temperature were the predictors of peak frequency in males and females, respectively, according to the best‐fitting LMMs (Table S3). Echolocation call duration in both sexes was not significantly influenced by monthly mean temperature (LMM: all *p* > 0.05; Figure 3 and Table 1). However, in males, peak frequency of echolocation calls was positively related to monthly mean precipitation (LMM: *β* = 0.35, *t* = 2.19, *p* = 0.034; Figure 3a and Table 1). In females, peak frequency of echolocation calls was negatively related to monthly mean temperature (LMM: *β* = −2.06, *t* = −4.53, *p* < 0.0001; Figure 3c and Table 1). SEM further revealed that these climatic variables had direct effects on peak frequency in males and females, respectively (males: *β* = 0.23, *p* = 0.036; females: *β* = −0.56, *p* < 0.0001; Figure 3b,d and Table S4), providing path‐based confirmation of the climatic effects identified by the LMMs.

**TABLE 1:** Effects of predictor variables on echolocation call parameters based on the best‐fitting linear mixed models.

Sex	Call parameter	Predictors	Estimate	*t*	*p*
Male	Peak frequency	(Intercept)	−0.07	−0.23	0.83
		**MMP**	**0.35**	**2.19**	**0.03**
Male	Call duration	(Intercept)	−0.01	−0.07	0.95
		MMT	−0.37	−2.01	0.08
Female	Peak frequency	(Intercept)	< 0.01	0.00	0.99
		**MMT**	**−2.06**	**−4.53**	**< 0.01**
Female	Call duration	(Intercept)	< 0.01	0.00	0.99
		MMT	0.54	0.27	0.79

*Note:* Data in bold are statistically significant.

Abbreviations: MMP, monthly mean precipitation; MMT, monthly mean temperature.

## 4 Discussion

In the present study, we investigated the factors underlying colony‐level variation in echolocation calls across eight colonies of *R. affinis* in the southern and northern regions of the Funiu Mountains. We found that male *R. affinis* emitted echolocation calls characterized by longer durations and lower peak frequencies during the resting phase compared to females. Moreover, the duration and peak frequency of echolocation calls exhibited pronounced variation among colonies. Although climatic factors did not significantly influence call duration, monthly mean precipitation and temperature exerted positive and negative effects on the peak frequency of echolocation calls in resting males and females, respectively. Collectively, these results support that ecological selection drives echolocation call divergence in CF bats at the landscape scale.

The monthly average precipitation was the sole significant predictor of the peak frequency of echolocation calls in male *R. affinis* . Three potential explanations may account for this phenomenon. First, sound attenuation in some anurans, birds, and mammals is more pronounced in humid habitats with high precipitation than in arid environments (Ey and Fischer 2009; Mutumi et al. 2016). Accordingly, rainfall may determine bat echolocation parameters by enhancing sound absorption in the atmosphere (Guillén et al. 2000; Mutumi et al. 2016). If increased atmospheric attenuation due to precipitation constitutes the primary selective pressure shaping inter‐colony variation in echolocation frequency of *R. affinis* , bats inhabiting habitats with greater rainfall would be expected to emit echolocation calls of lower frequency. Consequently, a negative relationship between monthly average precipitation and the peak frequency of echolocation calls would be observed. However, our results contradict this prediction. Second, elevated rainfall facilitates the proliferation of vegetation, likely leading to the formation of structurally complex habitats (Fensham et al. 2005; Xu et al. 2018). CF bats inhabiting complex habitats may produce higher frequency echolocation calls relative to those living in relatively open habitats since higher frequency signals improve the discrimination of nearby obstacles and insect prey, thereby enhancing echolocation performance in highly cluttered environments (Kalko and Schnitzler 1998; Schnitzler and Kalko 2001; Odendaal et al. 2014). According to this hypothesis, the peak frequency of echolocation calls in *R. affinis* would increase with rising NDVI values, but our findings do not support this prediction either. Third, heavy rainfall can generate broadband noise exceeding 70 dB when striking the ground, vegetation, and other surfaces (Scrimger 1985; Laville et al. 1991; Ma et al. 2022). Although the peak frequency of rain noise typically ranges from 10 to 15 kHz, its high‐frequency components can extend from 20 to 100 kHz (Figure 1b), overlapping with the first and second harmonics of *R. affinis* echolocation calls (Ma et al. 2022). In this case, rain noise may mask echolocation vocalizations of *R. affinis* as well as returning echoes from prey and surrounding objects (Schaub et al. 2008). To mitigate auditory masking effects, *R. affinis* inhabiting areas with higher precipitation may increase the frequency parameters of echolocation calls. Jiang, Metzner, et al. (2010) demonstrated that the peak frequency of echolocation calls emitted by *R. pusillus* was positively related to mean annual rainfall and relative humidity on a broad geographic scale. Greater horseshoe bats ( *R. ferrumequinum* ) changed the peak frequency and intensity of echolocation calls when exposed to synthetic noise simulating raindrops striking vegetation (Hage et al. 2013). These findings support the sensory drive hypothesis, suggesting that rainfall appears to shape echolocation call divergence in CF bats primarily through the masking effects of rain noise, rather than through atmospheric attenuation or selective pressures related to habitat complexity.

The monthly average temperature had significant negative effects on the peak frequency of echolocation calls in female *R. affinis* . This provides additional evidence in support of the sensory drive hypothesis. Previous acoustic studies have verified that echolocation calls in bats undergo atmospheric absorption during transmission through the air due to the absorption of sound energy by air molecules, which in turn restricts the effective range of echolocation (Lawrence and Simmons 1982; Goerlitz 2018). Atmospheric absorption is particularly evident for high‐frequency echolocation calls, especially under conditions of higher ambient temperature and relative humidity (Goerlitz 2018). Under this context, it is plausible that *R. affinis* colonies residing in habitats characterized by higher ambient temperature produce echolocation calls at lower frequencies to reduce atmospheric absorption. Similar findings have been documented in several other bat species. For example, mean annual temperature and relative humidity showed a significant interaction on the resting frequency of echolocation calls in Bushveld horseshoe bats ( *R. simulator* ) (Mutumi et al. 2016). When relative humidity was moderate to high, an increase in ambient temperature caused a reduction in the resting frequency of echolocation calls in *R. clivosus* (Jacobs et al. 2017). *H. armiger* reduced their peak frequency of echolocation calls in response to heightened atmospheric attenuation resulting from seasonal variations in ambient temperature, relative humidity, and atmospheric pressure (Wu et al. 2021). These findings indicate that ambient temperature drives echolocation call divergence in CF bats through its effect on atmospheric attenuation. Notably, sex‐specific responses to climate may reflect differences in reproductive state and behavioral ecology. In bats, adult females often rely on thermally favorable roosts and may avoid torpor during pregnancy and lactation, whereas males can show different roost use and thermoregulatory patterns (Turbill et al. 2020). Moreover, echolocation calls can contain sex‐specific information and may facilitate sex recognition and social communication. We therefore infer that the male‐specific response to precipitation and the female‐specific response to temperature in *R. affinis* may arise from sex‐specific ecological and social pressures rather than a single common mechanism. Further studies are needed to elucidate how climate differentially influences echolocation frequency in males and females, as this intriguing issue remains to be fully understood.

In many vocalizing mammals, larger species and individuals tend to possess thicker vocal cords in the larynx, which constrains them to produce relatively low‐frequency calls (Fitch and Hauser 2003; Taylor and Reby 2010). Indeed, an inverse relationship between echolocation call frequency and body size has been reported in some bats, both within and across species (Jacobs et al. 2007; Thiagavel et al. 2017; Russo et al. 2018; Luo et al. 2019; Castro et al. 2024). For instance, the peak frequency of echolocation calls in Greater leaf‐nosed bat ( *H. armiger* ) and Horsfield's leaf‐nosed bat ( *H. larvatus* ) from southern China exhibited a significant negative correlation with body size, with body mass showing a stronger association than head‐body length or forearm length (Zhang et al. 2000). Likewise, in Madagascar, Commerson's roundleaf bat ( *H. commersoni* ) displayed pronounced geographic variation in body size and echolocation call frequency, coupled with a strong inverse relationship between morphological and acoustic traits (Ramasindrazana et al. 2015). Comparative analyses in CF bats confirmed an overall negative scaling relationship between body mass and peak frequency of echolocation calls, reflecting morphological constraints imposed by the laryngeal‐nasal apparatus (López‐Cuamatzi et al. 2020), although the scaling slope varies by clades (Thiagavel et al. 2017; Castro et al. 2024). However, neither forearm length nor body mass was included in the best‐fitting LMM for predicting peak frequency of echolocation calls in male or female *R. affinis* . The best‐fitting SEM also showed that forearm length had no direct effect on peak frequency of echolocation calls in either sex. These results align with previous studies by Mutumi et al. (2016) and Jacobs et al. (2017), who reported that forearm length was not a significant predictor of resting echolocation frequency in *R. clivosus* , *R. simulator* , and *R. swinnyi* . The absence of a negative relationship between echolocation call frequency and forearm length or body mass in these bats implies that body size may not fully reflect their size and structural characteristics of the sound‐producing apparatus, at least at the intraspecific level. Further investigations are warranted to examine the morphological constraint hypothesis by analyzing the association between echolocation call frequency and anatomical characteristics of sound‐producing structures in CF bats.

In *R. affinis* , the peak frequency of echolocation calls exhibited a significant sexual difference, with males producing calls at lower frequencies than females (Figure 2d). Similar patterns of sexual dimorphism in call frequency have been reported across diverse bat species, including *R. rouxi* (Neuweiler et al. 1987), *R. monoceros* (Chen et al. 2009), *Eptesicus fuscus* (Grilliot et al. 2009), *R. mehelyi* (Schuchmann et al. 2012), *Saccopteryx bilineata* (Knornschild et al. 2012), *R. ferrumequinum* (Sun et al. 2013), and *H. pratti* (Fu et al. 2015). Consistent with these observations, Kazial and Masters (2004) found that *E. fuscus* individuals emitted vocalizations at higher rates in response to echolocation calls from male conspecifics compared with those from females. Additionally, Knornschild et al. (2012) demonstrated that the harem males of *S. bilineata* used echolocation calls to discriminate the sex of conspecifics, subsequently uttering territorial calls or courtship songs depending on the caller's identity. Furthermore, Pang et al. (2019) observed behavioral responses of *R. pusillus* to echolocation calls from colony members during the mating season, noting that these bats uttered more vocalizations in response to echolocation calls from males relative to females. Collectively, these findings suggest that echolocation calls provide reliable cues to the sex of the callers, thereby facilitating sex recognition and potentially influencing mate choice.

In summary, our landscape‐scale investigation demonstrated that the peak frequency of echolocation calls emitted by resting *R. affinis* was positively associated with monthly average precipitation in males and negatively associated with monthly average temperature in females, providing correlative support for the sensory drive hypothesis. These findings advance our understanding of sensory variation at fine spatial scales and suggest that local climatic conditions can shape acoustic divergence in horseshoe bats. Beyond sensory drive, previous studies have shown that genetic drift, cultural drift, and interspecific competition may also play roles in driving colony‐level variation in echolocation calls in some CF bats (Russo et al. 2007; Chen et al. 2009; Lin et al. 2015). Investigating the effects of these processes was outside the scope of this study. Future research should integrate acoustic, morphological, ecological, and genetic data to disentangle the adaptive and neutral processes underlying colony‐level call variation and to better understand acoustic adaptation.

## Author Contributions

**Dongge Guo:** conceptualization (lead), data curation (lead), formal analysis (lead), investigation (supporting), methodology (lead), visualization (lead), writing – original draft (lead), writing – review and editing (lead). **Lixian Wang:** conceptualization (equal), formal analysis (equal), investigation (equal), methodology (equal), project administration (equal), resources (equal), supervision (equal), validation (equal), visualization (equal), writing – original draft (supporting), writing – review and editing (equal). **Chong Zhang:** data curation (equal), investigation (equal), project administration (equal), supervision (equal), validation (equal). **Siyuan Dong:** data curation (equal), investigation (equal), project administration (equal), supervision (equal), validation (equal). **Fang Wang:** investigation (equal), methodology (equal), writing – review and editing (equal). **Bo Luo:** conceptualization (equal), data curation (equal), formal analysis (supporting), funding acquisition (lead), investigation (lead), methodology (equal), project administration (lead), resources (lead), supervision (lead), validation (lead), visualization (equal), writing – original draft (equal), writing – review and editing (lead). **Sen Liu:** conceptualization (lead), data curation (equal), formal analysis (equal), investigation (lead), methodology (lead), resources (equal), supervision (equal), validation (equal), visualization (equal), writing – review and editing (equal).

## Funding

This work was supported by the National Natural Science Foundation of China (32201265, 31870354, 32271561) and Research Fund for Doctoral Talents of Henan Normal University (QD20210237, QD2021003).

## Ethics Statement

The study was conducted in compliance with the law of China. Experimental procedures were in accordance with the Academic Ethics and Ethics Committee of Henan Normal University (permit ID: HNSD‐2024BS‐1105 and HNSD‐2024BS‐1230).

## Conflicts of Interest

The authors declare no conflicts of interest.

## Supporting information


**Table S1:** Roost locations and collected data of different colonies.
**Table S2:** Statistical comparisons among different colonies of *Rhinolophus affinis* .
**Table S3:** The first five alternative linear mixed models of echolocation call parameters.
**Table S4:** The alternative path models of peak frequency of echolocation calls in *Rhinolophus affinis* .

## Data Availability

Data are available as Supporting Information.
