# Supplementary material for: Rainfall and Temperature Explain Colony Variation in Echolocation Calls of the Intermediate Horseshoe Bats (Rhinolophus affinis)
Source: Ecol Evol. 2026 Apr 8;16(4):e73485. doi: 10.1002/ece3.73485 (PMC13061745; doi:10.1002/ece3.73485)
Supplement: Supplementary file 1 — Table S1: Roost locations and collected data of different colonies. Table S2: Statistical comparisons among different colonies of Rhinolophus affinis . Table S3: The first five alternative linear mixed models of echolocation call parameters. Table S4: The alternative path models of peak frequency of echolocation calls in Rhinolophus affinis . [file ECE3-16-e73485-s001.docx]

**Supplementary Material**

**Table S1** Roost locations and collected data of different colonies

| ID | Cave name | City | N | Sex | PF  /kHz | CD  /ms | FL  /mm | BM  /g | Alt.  /m | MMT  /℃ | MMP  /mm | NDVI |
| --- | --- | --- | --- | --- | --- | --- | --- | --- | --- | --- | --- | --- |
| C1 | Tianxin Cave | Nanyang | 12  9 | Male  Female | 81.47 ± 0.42  82.03 ± 0.48 | 38.00 ± 5.76  32.76 ± 8.47 | 56.12 ± 0.85  55.46 ± 0.89 | 18.40 ± 0.66  23.39 ± 1.16 | 344 | 14.85 | 68.75 | 0.24 |
| C2 | Yunhua Cave | Nanyang | 7  12 | Male  Female | 81.43 ± 0.49  82.03 ± 0.35 | 28.24 ± 4.12  31.32 ± 6.18 | 55.04 ± 0.95  55.49 ± 0.81 | 16.90 ± 1.35  22.80 ± 3.10 | 418 | 15.09 | 69.00 | 0.23 |
| C3 | Jiulong Cave | Nanyang | 10  0 | Male  Female | 80.60 ± 0.62 | 37.36 ± 4.34 | 54.62 ± 1.89 | 18.34 ± 2.04 | 896 | 13.09 | 65.67 | 0.25 |
| C4 | Longsangong Cave | Sanmenxia | 14  12 | Male  Female | 81.93 ± 0.53  82.31 ± 0.40 | 31.80 ± 6.09  32.00 ± 4.73 | 54.94 ± 0.93  55.17 ± 1.15 | 18.01 ± 1.44  18.14 ± 1.27 | 584 | 14.37 | 63.17 | 0.25 |
| C5 | Shuilian Cave | Luoyang | 5  0 | Male  Female | 79.87 ± 0.26 | 38.07 ± 2.52 | 55.37 ± 0.72 | 19.45 ± 0.83 | 517 | 13.23 | 59.00 | 0.22 |
| C6 | Xianren Cave | Nanyang | 10  12 | Male  Female | 82.70 ± 0.40  82.69 ± 0.41 | 33.40 ± 5.45  30.32 ± 6.03 | 56.83 ± 0.70  55.98 ± 0.96 | 18.84 ± 1.07  19.81 ± 1.04 | 528 | 13.79 | 67.33 | 0.24 |
| C7 | Xiapu Mine | Luoyang | 12  1 | Male  Female | 81.04 ± 0.15  82.93 | 40.92 ± 4.43  30.91 | 55.10 ± 1.01  56.57 | 19.60 ± 1.96  19.23 | 848 | 12.23 | 63.00 | 0.26 |
| C8 | Kafang Mine | Luoyang | 12  1 | Male  Female | 80.44 ± 0.79  83.00 | 38.61 ± 2.59  33.31 | 55.42 ± 1.30  55.53 | 18.03 ± 1.21  16.15 | 1061 | 11.63 | 66.75 | 0.25 |

PF: Peak frequency. CD: Call duration. FL: Forearm length. BM: Body mass. Alt.: Altitude. MMT: Monthly mean temperature. MMP: Monthly mean precipitation. NDVI: Normalized difference vegetation index.

**Table S2** Statistical comparisons among different colonies of *Rhinolophus affinis*

| Call duration | C2 | C3 | C4 | C5 | C6 | C7 | C8 |
| --- | --- | --- | --- | --- | --- | --- | --- |
| C1 | 0.05 | 0.99 | 0.29 | 0.99 | 0.29 | 0.37 | 0.93 |
| C2 | – | **0.03** | 0.98 | 0.11 | 0.99 | **<0.001** | **<0.01** |
| C3 |  | – | 0.17 | 1 | 0.16 | 0.94 | 0.99 |
| C4 |  |  | – | 0.34 | 1 | **<0.001** | 0.05 |
| C5 |  |  |  | – | 0.33 | 0.99 | 1 |
| C6 |  |  |  |  | – | **<0.001** | 0.05 |
| C7 |  |  |  |  |  | – | 0.99 |
| Peak frequency | C2 | C3 | C4 | C5 | C6 | C7 | C8 |
| C1 | 0.99 | **<0.001** | 0.30 | **<0.001** | **<0.001** | 0.18 | **<0.001** |
| C2 | – | **<0.001** | 0.71 | **<0.001** | **<0.001** | 0.06 | **<0.001** |
| C3 |  | – | **<0.001** | 0.31 | **<0.001** | 0.24 | 1 |
| C4 |  |  | – | **<0.001** | **0.01** | **<0.001** | **<0.001** |
| C5 |  |  |  | – | **<0.001** | **<0.001** | 0.19 |
| C6 |  |  |  |  | – | **<0.001** | **<0.001** |
| C7 |  |  |  |  |  | – | 0.25 |

C1: Tianxin Cave; C2: Yunhua Cave; C3: Jiulong Cave; C4: Longsangong Cave; C5: Shuilian Cave; C6: Xianren Cave; C7: Xiapu Mine; C8: Kafang Mine. Data in bold are statistically significant.

**Table S3** The first five alternative linear mixed models of echolocation call parameters

| Sex | Dependent variable | Models | Interccpt | *Df* | AICc | ΔAICc |
| --- | --- | --- | --- | --- | --- | --- |
| Male | Peak frequency | MMP | -0.071 | 4 | 172.40 | 0.00 |
| Male | Peak frequency | MMT | -0.108 | 4 | 172.77 | 0.37 |
| Male | Peak frequency | Altitude + NDVI | -0.007 | 5 | 173.35 | 0.95 |
| Male | Peak frequency | Altitude + MMP | -0.099 | 5 | 173.92 | 1.53 |
| Male | Call duration | MMT | -0.013 | 4 | 222.06 | 0.78 |
| Male | Call duration | Altitude | -0.004 | 4 | 222.50 | 1.22 |
| Male | Call duration | MMP | -0.038 | 4 | 223.25 | 1.96 |
| Male | Call duration | MMT + Altitude | -0.010 | 5 | 224.54 | 3.25 |
| Male | Call duration | MMP + Altitude | -0.016 | 5 | 224.93 | 3.64 |
| Female | Peak frequency | MMT | <0.001 | 4 | 128.41 | 0.00 |
| Female | Peak frequency | MMT + Altitude | -0.005 | 5 | 131.70 | 3.29 |
| Female | Peak frequency | Altitude | -0.011 | 4 | 131.97 | 3.56 |
| Female | Peak frequency | MMT+NDVI | -0.003 | 5 | 132.63 | 4.22 |
| Female | Call duration | MMT | <0.001 | 4 | 145.25 | 4.30 |
| Female | Call duration | NDVI | <0.001 | 4 | 145.26 | 4.31 |
| Female | Call duration | FL | <0.001 | 4 | 145.29 | 4.34 |
| Female | Call duration | Altitude | <0.001 | 4 | 145.31 | 4.36 |
| Female | Call duration | MMP | <0.001 | 4 | 145.32 | 4.37 |
| **Additional analysis: Body mass was used as the alternative proxy for body size.** | | | | | | |
| Male | Peak frequency | MMP | -0.071 | 4 | 172.40 | 0.00 |
| Male | Peak frequency | MMT | -0.108 | 4 | 172.77 | 0.37 |
| Male | Peak frequency | Altitude + NDVI | -0.007 | 5 | 173.35 | 0.95 |
| Male | Peak frequency | Altitude + MMP | -0.099 | 5 | 173.92 | 1.53 |
| Male | Call duration | MMT | -0.013 | 4 | 222.06 | 0.78 |
| Male | Call duration | Altitude | -0.004 | 4 | 222.50 | 1.22 |
| Male | Call duration | MMP | -0.038 | 4 | 223.25 | 1.96 |
| Male | Call duration | Altitude + MMT | -0.010 | 5 | 224.54 | 3.25 |
| Male | Call duration | Altitude + MMP | -0.016 | 5 | 224.93 | 3.64 |
| Female | Peak frequency | MMT | <0.001 | 4 | 128.41 | 0.00 |
| Female | Peak frequency | MMT + Altitude | -0.005 | 5 | 131.70 | 3.29 |
| Female | Peak frequency | Altitude | -0.011 | 4 | 131.97 | 3.56 |
| Female | Peak frequency | MMT + BM | <0.001 | 5 | 132.24 | 3.83 |
| Female | Peak frequency | MMT + NDVI | -0.003 | 5 | 132.63 | 4.22 |
| Female | Call duration | MMT | <0.01 | 4 | 145.25 | 4.30 |
| Female | Call duration | NDVI | <0.01 | 4 | 145.26 | 4.31 |
| Female | Call duration | BM | <0.01 | 4 | 145.28 | 4.33 |
| Female | Call duration | Altitude | <0.01 | 4 | 145.31 | 4.36 |
| Female | Call duration | MMP | <0.01 | 4 | 145.32 | 4.37 |

MMT: Monthly mean temperature. MMP: Monthly mean precipitation. FL: Forearm length. BM: Body mass. NDVI: Normalized difference vegetation index.

**Table S4** The alternative path models of peak frequency of echolocation calls in *Rhinolophus affinis*

| Sex | Endogenous variable | Models | AICc |
| --- | --- | --- | --- |
| Male | Peak frequency | Full model | 449.00 |
| Male | Peak frequency | MMT | 447.16 |
| Male | Peak frequency | MMP | 463.58 |
| Male | Peak frequency | FL | 464.63 |
| Male | Peak frequency | **MMT + MMP** | **446.00** |
| Male | Peak frequency | MMT + FL | 446.75 |
| Male | Peak frequency | MMP + FL | 464.09 |
| Female | Peak frequency | Full model | 260.34 |
| Female | Peak frequency | **MMT** | **257.56** |
| Female | Peak frequency | MMP | 272.87 |
| Female | Peak frequency | FL | 272.69 |
| Female | Peak frequency | MMT + MMP | 260.34 |
| Female | Peak frequency | MMT + FL | 257.57 |
| Female | Peak frequency | MMP + FL | 272.81 |

MMT: Monthly mean temperature. MMP: Monthly mean precipitation. FL: Forearm length. Data in bold represent the best-fitting model.
